# Multivitamin Use and Serum Vitamin B12 Concentrations in Older-Adult Metformin Users in REGARDS, 2003-2007

**DOI:** 10.1371/journal.pone.0160802

**Published:** 2016-08-11

**Authors:** Vijaya Kancherla, Joshua V. Garn, Neil A. Zakai, Rebecca S. Williamson, Winn T. Cashion, Oluwaseun Odewole, Suzanne E. Judd, Godfrey P. Oakley

**Affiliations:** 1 Department of Epidemiology, Rollins School of Public Health, Emory University, Atlanta, Georgia, United States of America; 2 Departments of Medicine and Pathology, College of Medicine, University of Vermont College, Burlington, Vermont, United States of America; 3 School of Public Health, University of Alabama at Birmingham, Birmingham, Alabama, United States of America; Baylor College of Medicine, UNITED STATES

## Abstract

Metformin, an insulin-sensitizing drug, is a first line treatment for type 2 diabetes. Long-term use of metformin has been associated with subsequent reductions in vitamin B12 concentrations. The objective of our study was to determine whether metformin use is associated with lower serum vitamin B12 concentrations in older adults, and whether concurrent use of multivitamins modifies this association. We examined 2,510 participants aged 50 years and over, participating in the national population-based Reasons for Geographic And Racial Differences in Stroke (REGARDS) Study. Multivariable linear and logistic regression models were used to assess associations between multivitamin use and serum vitamin B12 concentrations. We estimated adjusted odds ratios (aOR)s and confidence intervals (CI)s. Results were stratified by three metformin/diabetes sub-groups: 1) participants with diabetes who were metformin users; 2) participants with diabetes who were not metformin users; and 3) participants without diabetes. We found that diabetic metformin users had significantly lower geometric mean serum B12 concentrations (409 pmol/L) than the group with diabetes not taking metformin (485 pmol/L; *P*<0.01), and the group without diabetes (445 pmol/L; *P* = 0.02). The geometric mean serum B12 concentrations were greater for multivitamin users (509 pmol/L) compared to those who did not use multivitamins (376 pmol/L; *p*<0.01). Among the participants with diabetes who were on metformin therapy, multivitamin use was associated with geometric mean serum vitamin B12 concentrations that were 50% (or 161 pmol/L) higher, compared to those not using multivitamins. Among metformin users, multivitamin use was associated with lower prevalence of combined low and borderline vitamin B12 concentrations (aOR = 0.14; 95% CI = 0.04, 0.54) compared to those not using multivitamins. In conclusion, metformin use was associated with lower geometric mean serum vitamin B12 concentrations among diabetic older adults compared to their counterparts. Concurrent multivitamin use may potentially protect against low or borderline vitamin B12 concentrations in long-term metformin users. Additional research is needed to further examine this association as low or borderline vitamin B12 concentrations can be preventable, or treatable if detected at an early stage, in long-term metformin users.

## Introduction

Metformin, an insulin-sensitizing drug, is a first line treatment for type 2 diabetes [1,2]. Currently, an estimated 120 million people use metformin worldwide [3]. Metformin is also being considered for other non-diabetic indications in older adults, including cancer [3,4]. However, long-term use of metformin has been associated with vitamin B12 deficiency in about 6–30% of its users [5,6–15]. A recent meta-analysis of several published cohort studies and randomized clinical trials further confirmed that metformin use is significantly associated with an increase in the occurance of vitamin B12 deficiency [16]. However, an important consideration is that vitamin B12 deficiency can be measured using several markers, and there is no consensus on the clinical definition of B12 deficiency [17–21]. None of the larger studies had examined functional biomarkers of vitamin B12 deficiency (including methylmalonic acid or total homocysteine) in addition to serum concentrations of vitamin B12 [11,13].

The Institute of Medicine (IOM), Food Nutrition Board (FNB) recommends that all persons 50 years and over consume a daily dose of 2.4 μg of synthetic vitamin B12 from vitamin supplements or from food fortified with vitamin B12 [22]. Several studies have shown that pharmacological doses of vitamin B12 supplements (a recommended dose of 1000 μg per day) correct vitamin B12 deficiency in cases arising due to inadequate vitamin B12 intake or its malabsorption [23–25]. A potentially protective association has been reported between low dose vitamin B12 supplement intake (such as a common multivitamin with 6–25 μg of vitamin B12) and biochemical B12 deficiency and low serum B12 concentrations [23,26].

While the effect of metformin and vitamin B12 supplements on serum B12 concentrations are separately well researched, it is unknown whether multivitamins with low dose vitamin B12 (6–25 μg of vitamin B12) might attenuate serum B12 deficiency among older patients with diabetes who are receiving metformin therapy. A review by Valdes-Ramos et al. (2015) suggested vitamin supplementation as an intervention to address low vitamin B12 status among diabetic patients who have a history of long-term use of metformin, although the evidence supporting this was mixed [27].

Reinstatler et al. (2012), in their large population-based study using 1999–2006 National Health and Nutrition Examination Survey (NHANES) among adults of ≥60 years of age, found that vitamin B12 supplementation (doses up to 6 μg per day) did not reduce the prevalence odds of biochemical B12 deficiency (<149 pmol/L) or borderline deficiency (149–221 pmol/L) among subjects with diabetes and taking metformin [13]. Pflipsen et al. (2009), using similar methods as Reinstatler et al. (2012), but a different definition of biochemical B12 deficiency (B12 concentration <100 pg/mL) and intermediate B12 deficiency (B12 concentrations 100–350 pg/mL, while accounting for high methylmalonic acid >243 nmol/L and/or high homocysteine > 11.9 nmol/L), found multivitamin use was associated with significantly lower odds of serum B12 deficiency in a cross-sectional sample of adults aged 45 years of older [10,13]. Both the studies simultaneously controlled for metformin use and multivitamin use in their analyses; but neither examined if such associations were further modulated by each other.

The aim of this study was to determine in a descriptive, cross-sectional analysis, whether metformin use was associated with reduction in serum vitamin B12 concentration in older adults, and whether concurrent use of multivitamin supplements modified this association among diabetic and non-diabetic patients using a large sample of participants enrolled in the REasons for Geographic And Racial Differences in Stroke (REGARDS) study. Our overall goal was to generate population-based findings to assess if there is an association between multivitamin use and low concentrations of vitamin B12 among older adults with diabetes and long-term metformin use, and prompt prospective and interventional studies that can explore appropriate prevention strategies.

## Materials and Methods

The REGARDS study is a national population-based cohort study on stroke risk factors among a biracial sample of 30,239 black and white adults aged 45 years and older. Participants were recruited from a nationwide list of more than 250 million individuals living in the contiguous United States using commercial lists (Gerysis, Daly City, CA, USA) between January 2003 and October 2007. The study recruited participants with an equal representation of sex and race; however, participants from the Southeastern States (AL, AR, GA, LA, MS, NC, SC, and TN) were oversampled, where 50% of the cohort was selected from the stroke belt to study the risk of heart disease. Additional details of the study are published elsewhere [28].

All enrolled participants completed a computer-assisted telephone interview to provide demographic information and medical history, followed by an in-home physical examination to measure cardiovascular risk factors, using standardized protocols. Follow up telephone interviews were conducted at six month intervals for surveillance of cardiovascular and general health status [28]. The study was approved by the institutional review board at each of the participating institution, and an external monitoring board appointed by the funding agency.

**Fig 1:**
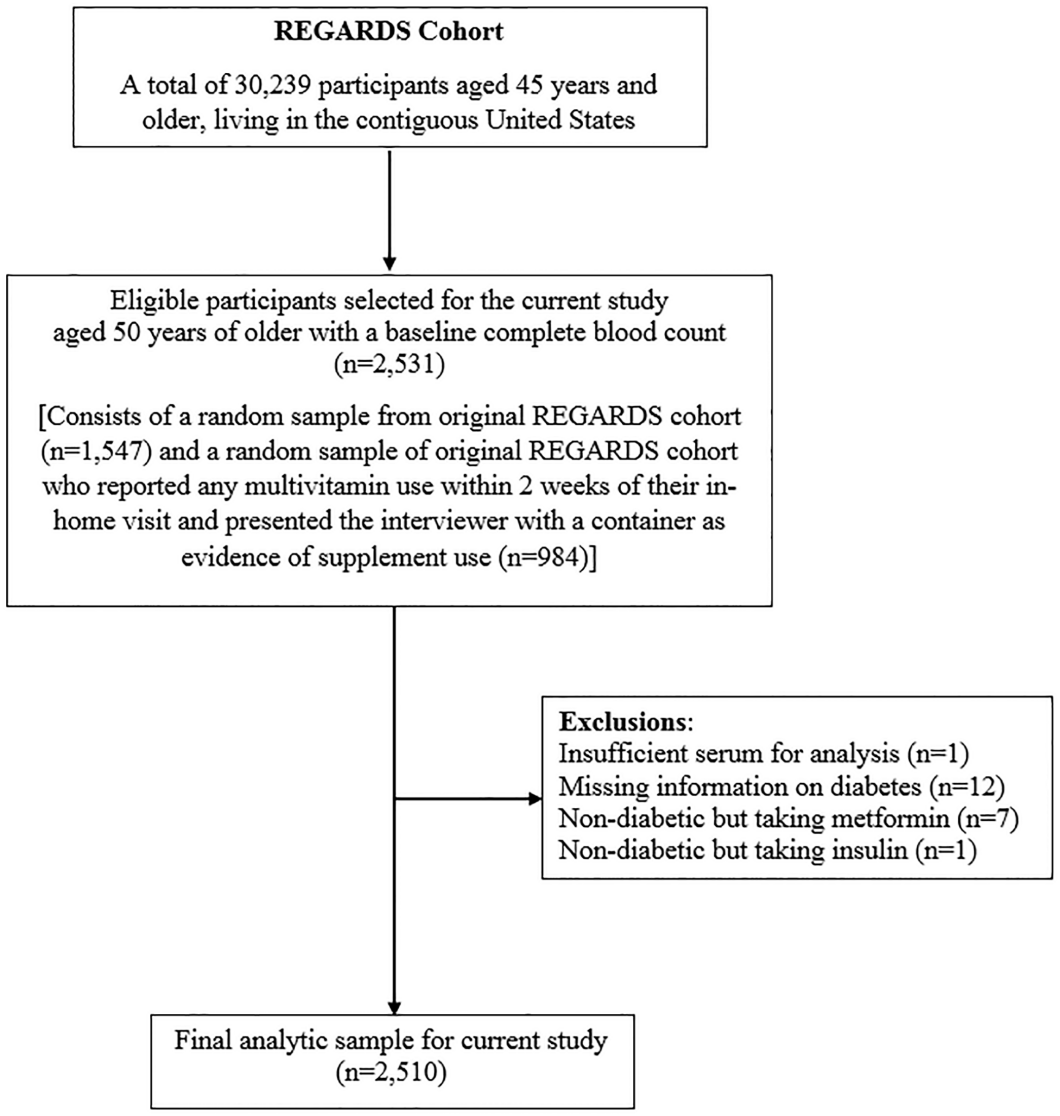
Subject selection criteria—REasons for Geographic And Racial Differences in Stroke, 2003–2007.

For our current analysis, we selected 2,531 participants aged 50 years and older, a sub- sample of those participating in the REGARDS (Fig 1). This sub-sample included a random sample of 1,547 individuals who had baseline data for complete blood count. To have an adequate number of multivitamin users, an additional 984 participants were randomly sampled from those who reported any use of multivitamin supplements within 2 weeks of their in-home visit and presented the interviewer with a container as evidence of supplement use. Because we oversampled multivitamin users, our sample may not be representative of the original REGARDS target population.

Serum vitamin B12 concentrations were measured on all participants at the time of enrollment in the REGARDS using the Elecsys Vitamin B12 assay (Roche Diagnostics, Indianapolis, IN) at the University of Vermont (www.med.uvm.edu/lcbr/). For our analysis, we defined low B12 concentration as <148 pmol/L. Borderline B12 status was established as concentrations between 148–221 pmol/L, and normal as >221 pmol/L. Our rationale for categorizing vitamin B12 concentrations are consistent with previously published cut points [13]. We did not have information on other functional biomarkers (methylmalonic acid and/or homocysteine) to confirm vitamin B12 deficiency among participants with borderline measures of vitamin B12 concentrations.

We examined metformin use as the primary exposure of interest. Data on metformin use was collected in the REGARDS based on a “pill bottle” inspection during the in-home medication inventory procedure. Specifically, the pill bottle inspection method consisted of the participants showing a trained health professional any medications (including over the counter medications and supplements) that they had used at least once during two weeks prior to the in-home interviews. We coded metformin exposure as a dichotomous variable (use / no use). Type 2 diabetes status was self-reported by the participants during the interview and examined in the current analysis as a dichotomous variables (yes / no).

Use of multivitamins, insulin, proton pump inhibitors (PPI), H2 receptor antagonists (H2RA), antacids, or angiotensin-converting-enzyme (ACE) inhibitors were classified dichotomously (use / no use) and were also based on a “pill bottle” inspection that took place during the in-home medication inventory.

Age, race (black/white), and sex were self-reported on the baseline telephone survey. REGARDS residence region of participants, based on their physical address, was categorized as either living in the stroke belt (an area of high stroke incidence located in North Carolina, South Carolina, Georgia, Tennessee, Mississippi, Alabama, Louisiana, Arkansas), in the stroke buckle (an area of even higher stroke incidence located in the coastal plains of North Carolina, South Carolina, and Georgia), or in any of the other regions of the United States [28]. Smoking status was self-reported by the participant as either currently smoking or not smoking. Participant’s alcohol use was also self-reported and was classified as no use (0 drinks/week), moderate use (1–7 drinks/week for women and 1–14 drinks/week for men), or heavy use (7+ drinks/week for women and 14+ drinks/week for men). Height, weight, and body mass index (BMI) (calculated as weight in kilograms divided by height in meters squared), were all measured and recorded during the in-home visit by a trained health professional (Examination Management Systems INC, Scottsdale, AZ).

### Statistical Analysis

We estimated means and proportions for various demographic and clinical characteristics, and compared them between those who reported using multivitamins and those who did not. Similar comparisons were conducted between participants with and without diabetes; among diabetics, we compared those with and without metformin use. Tests of significance for differences were examined using on Chi square test or Fisher exact test for categorical variables, and independent sample *t* tests for continuous variables.

We conducted univariable analysis to examine the distribution of data for serum B12 concentration in our study sample. Upon determining that data were not normally distributed, we log-transformed the serum B12 concentrations to meet the normal distribution assumption to carry out the linear regression procedure. Geometric means (and 95% confidence intervals) for serum B12 concentrations were estimated. We used linear regression modelling to examine the association between log-transformed serum B12 concentration (as a continuous dependent variable) and multivitamin use (as categorical predictor variable). Similarly, we examined the association between log-transformed serum B12 concentration and diabetes/metformin use status (categorical). We used multiple linear regression modelling to examine the association between serum B12 concentration and multivitamin use, and diabetes/metformin use status, while controlling for potential confounders. All confounders were selected based on *a priori* criterion, using previously published studies [10,13,29]. Beta coefficients and standard errors were estimated for the main predictor variables. All models were tested for significance using the F test.

Using logistic regression analysis, we calculated unadjusted (uOR) and adjusted odds ratios (aOR), and 95% confidence intervals (95% CI) to quantify the association between multivitamin use and different categories of low vitamin B12 concentration (OR_low_) (Low: <148 pmol/L vs. Normal: >221 pmol/L) and for borderline concentration (OR_bord_) (Borderline: 148–221 pmol/L vs. Normal >221 pmol/L). The association was further examined by different strata of diabetes and metformin exposure sub-groups, including: 1) participants with diabetes who were metformin users; 2) participants with diabetes who were not metformin users, and; and 3) participants without diabetes. Confounders were selected on *a priori* criterion from previously published studies [10,13,29]. The final logistic regression model adjusted for age, race, sex, geographic region, BMI, smoking, alcohol use and insulin. All analyses were conducted using SAS 9.3 (SAS Institute, Cary, NC).

## Results

Of the 2531 participants that were sampled for our analysis from the REGARDS study, 2510 (99.2%) met inclusion criteria (Fig 1). Of those excluded, one had insufficient serum for assessing vitamin B12 concentration, twelve had missing information on diabetes-related questions, seven were non-diabetic but taking metformin, and one was non-diabetic but taking insulin.

**Fig 2:**
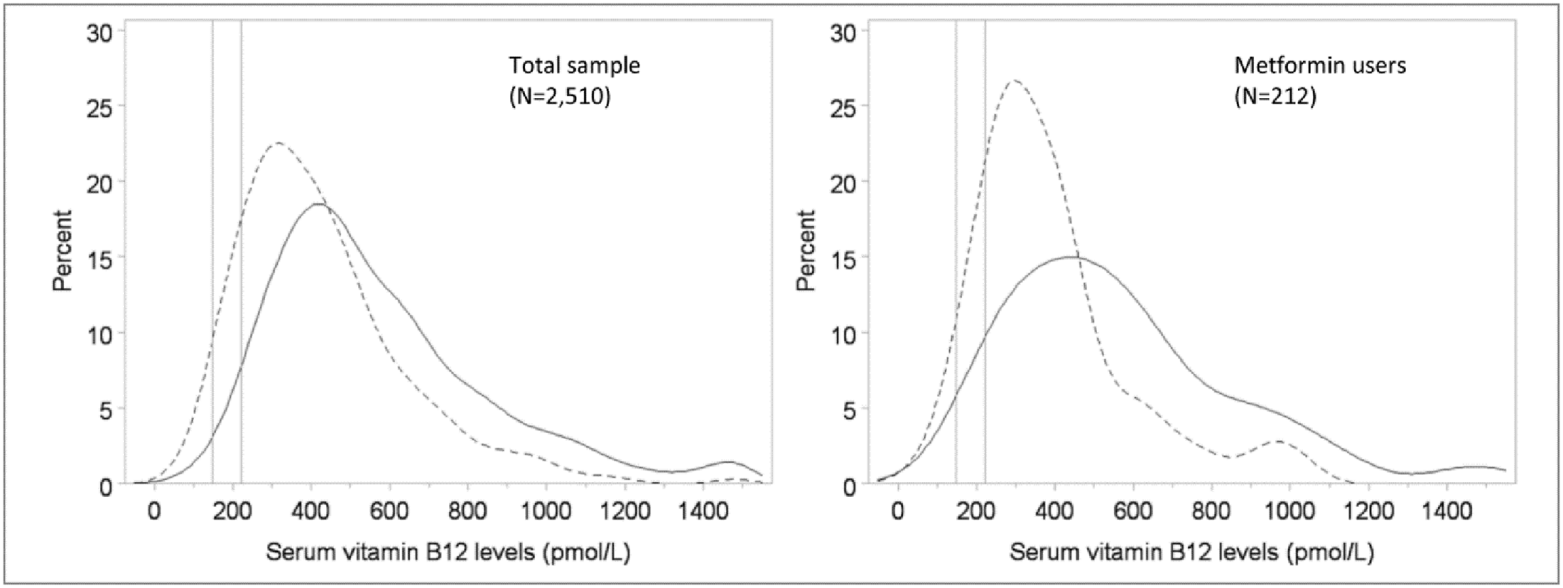
Distribution of serum vitamin B12 concentrations by multivitamin use—REasons for Geographic And Racial Differences in Stroke, 2003–2007. Dotted line represents the distribution of B12 concentrations for non-multivitamin users; Vertical line at 148 pmol/L and 221 pmol/L represent the cutoffs for low vitamin B12 concentration, and borderline B12 concentration, respectively.

We compared the geometric mean serum B12 concentrations between participants with and without multivitamin use, and found that multivitamin users had a significantly higher geometric mean serum B12 concentration (509 pmol/L) compared to those who did not take multivitamins (376 pmol/L) *P*<0.01) (Table 1). Additionally, vitamin B12 concentration was dichotomized as sub-normal vs. normal (≤221 pmol/L vs. >221 pmol/L); only 4% of multivitamin users had a sub-normal concentration of vitamin B12 compared to 15% of non-multivitamin users (*P*<0.01). Comparison of demographic and clinical characteristics between those with and without multivitamin use revealed that multivitamin users were less likely to have diabetes (*P*<0.01) or take metformin (*P*<0.01) compared to non-multivitamin users. Multivitamin use was also significantly associated with older age, lower BMI, being white, being female, not smoking, taking antacids, and region of residence (Table 1). Fig 2 shows the distribution of serum B12 concentrations by multivitamin use for both the overall study population, and separately by metformin use status.

**Table 1:** Demographic and Clinical Characteristics of Older Adults (50 years or older) by Multivitamin use and by Diabetes/Metformin Use Status—REasons for Geographic And Racial Differences in Stroke (N = 2510), 2003–2007.

Characteristics	Multivitamin Use			Diabetes/Metformin				
	Yes n (%)	No n (%)	*P* value*	Diabetes			No Diabetes	
				Metformin n (%)	No metformin n (%)	*P* value^†^	No Metformin	*P* value^‡^
Total Participants, *n*	1431	1079		212	331		1967	
Multivitamin Use, *n (%)*	-	-		99 (47%)	173 (52%)	0.21	1160 (59%)	<0.01
Serum B12 (pmol/L), mean^§^ (SD)	509 (1.6)	376 (1.7)	<0.01	409 (1.7)	485 (1.6)	<0.01	445 (1.7)	0.02
Low B12 (<148 pmol/L), *n (%)*	14 (1%)	37 (3%)	<0.01	5 (2%)	4 (1%)	0.14	42 (2%)	0.66
Borderline B12 (148–221 pmol/L), *n (%)*	40 (3%)	119 (11%)		17 (8%)	15 (5%)		127 (6%)	
Metformin use, *n (%)*	99 (7%)	113 (10%)	<0.01	-	-		-	
Type 2 Diabetes, *n (%)*	272 (19%)	271 (25%)	<0.01	-	-		-	
Age, mean (SD)	66.2 (8.8)	65.1 (8.7)	<0.01	65 (8.2)	67 (8.5)	<0.01	66 (8.8)	0.28
Race—Black, *n (%)*	421 (29%)	462 (43%)	<0.01	102 (48%)	179 (54%)	0.18	602 (31%)	<0.01
Female, *n (%)*	950 (66%)	653 (61%)	<0.01	125 (59%)	197 (60%)	0.90	1281 (65%)	0.07
Lives in Belt, *n (%)*	498 (35%)	358 (33%)	0.02	79 (37%)	116 (35%)	0.87	661 (34%)	0.54
Lives in Buckle, *n (%)*	342 (24%)	311 (29%)		54 (26%)	87 (26%)		512 (26%)	
Lives in Other region, *n (%)*	591 (41%)	410 (38%)		79 (37%)	128 (39%)		794 (40%)	
Body Mass Index (kg/m^2)^, mean (SD)^¶^	28.8 (6.2)	29.6 (6.2)	<0.01	32.3 (6.3)	31.9 (7.1)	0.49	28.4 (5.8)	<0.01
Currently smoking^||^, *n (%)*	146 (10%)	162 (15%)	<0.01	28 (13%)	38 (12%)	0.56	242 (12%)	0.72
Heavy alcohol use^#^	43 (3%)	41 (3.9%)	0.12	1(0.5%)	5 (2%)	0.23**	78 (4%)	<0.01
Moderate Alcohol Use^#^, *n (%)*	505 (36%)	342 (32%)		42 (20%)	83 (25%)		722 (37%)	
No Alcohol Use^#^, *n (%)*	859 (61%)	678 (64%)		164(79%)	239 (73%)		1134 (59%)	
Insulin Use, *n (%)*	43 (4%)	43 (4%)	0.18	18 (8%)	68 (21%)	<0.01	-	-
Proton pump inhibitor use, *n (%)*	244 (17%)	188 (17%)	0.81	42 (20%)	62 (19%)	0.76	328 (17%)	0.25
H_2_ receptor use, *n (%)*	65 (5%)	37 (3%)	0.16	10 (5%)	17 (5%)	0.83	75 (4%)	0.52
Antacid use, *n (%)*	113 (8%)	36 (3%)	<0.01	4 (2%)	12 (4%)	0.24	133 (7%)	<0.01
ACE inhibitor use, *n (%)*	325 (23%)	253 (23%)	0.66	96 (45%)	145 (44%)	0.74	337 (17%)	<0.01

*Comparing multivitamin users to non-multivitamin users.

^†^Comparing metformin users to those who are not metformin users with diabetes.

^‡^Comparing metformin users to those without diabetes.

^§^Geometric mean.

^¶^21 Missing values.

^||^7 Missing values.

^#^42 Missing values for alcohol variable; none = 0 drinks/week; moderate = 1–7 drinks/week for women and 1–14 drinks/week for men; heavy = 7+ drinks/week for women and 14+ drinks/week for men.

**Fisher’s exact test.

ACE, Angiotensin Converter Enzyme; SD, Standard Deviation.

Overall, 21.6% (n = 543) of the participants had diabetes, and among them, 39% (n = 212) were on a metformin therapy (Table 1). We compared geometric mean serum B12 concentrations (both as continuous and categorical measures) and demographic and clinical characteristics among participants by their diabetes and metformin use status, and found that diabetic metformin users had a significantly lower geometric mean serum B12 concentrations (409 pmol/L) than the group with diabetes not taking metformin (485 pmol/L; *P*<0.01), and the group without diabetes (445 pmol/L; *P* = 0.02). Metformin users were the least likely to have taken a multivitamin in the previous two weeks, with 47% of the metformin users with diabetes having taken a multivitamin, 52% of the non-metformin users with diabetes having taken a multivitamin, and 59% of those without diabetes having taken a multivitamin (*P*<0.01). Furthermore, of the metformin users, only 18% (4 out of 22) of those who were classified as having either low or borderline vitamin B12 concentrations had reported taking a multivitamin in the previous two weeks (data not shown). Among those with diabetes, metformin use was significantly associated with decreasing age, and decreased insulin use. When comparing metformin users to those without diabetes, metformin use was significantly associated with being black, increased BMI, decreased alcohol use, decreased antacid use, and increased ACE inhibitor use (Table 1).

**Fig 3:**
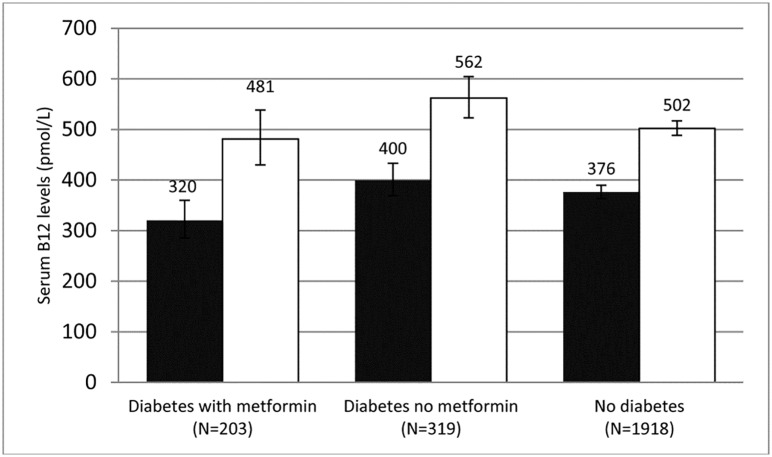
Modeled geometric mean serum vitamin B12 concentrations and 95% confidence intervals stratified by diabetes/metformin status—REasons for Geographic And Racial Differences in Stroke, 2003–2007. Adjusted for race, gender, geographic region, body mass index, alcohol use, smoking, insulin use, and age (all at their population mean levels). White = Multivitamin user; Black = Non-multivitamin user. *Totals are reduced due to missing data.

In the multiple linear regression analyses, participants with diabetes taking both metformin and multivitamins showed a significant increase in log serum B12 concentrations (β = 0.407; standard error (SE) = 0.068; *P*<0.01), compared to those not taking multivitamins, controlling for age, race, sex, geographic region, BMI, smoking, alcohol use and insulin use. Fig 3 shows the geometric means of vitamin B12 concentrations for each of the diabetic treatment groups (metformin vs. no metformin) and those without diabetes and without metformin, stratified by multivitamin use, adjusting for aforementioned covariables. Among those with diabetes taking metformin, the mean serum B12 concentration was 481 pmol/L (95% CI = 430–538 pmol/L) for multivitamin users compared to 320 pmol/L (95% CI = 285–360 pmol/L) for non-multivitamin users. Among those with diabetes not taking metformin, the mean serum B12 concentration in the non-metformin diabetes group was 562 pmol/L (95% CI = 523–604 pmol/L) for multivitamin users compared to 400 pmol/L (95% CI = 369–433 pmol/L) for non-multivitamin users. Among those without diabetes, the mean serum B12 concentration was 502 pmol/L (95% CI = 488–517 pmol/L) for multivitamin users compared to 376 pmol/L (95% CI = 363–390 pmol/L) for non-multivitamin users. Comparing above three cases, an increase in the geometric mean serum B12 concentration was of highest magnitude (50%) in the group with diabetes, where metformin and multivitamin use was concurrent. The other groups showed a lower percent increase in their B12 concentration, 41% and 34%, respectively, depending on whether or not they used multivitamins.

**Fig 4:**
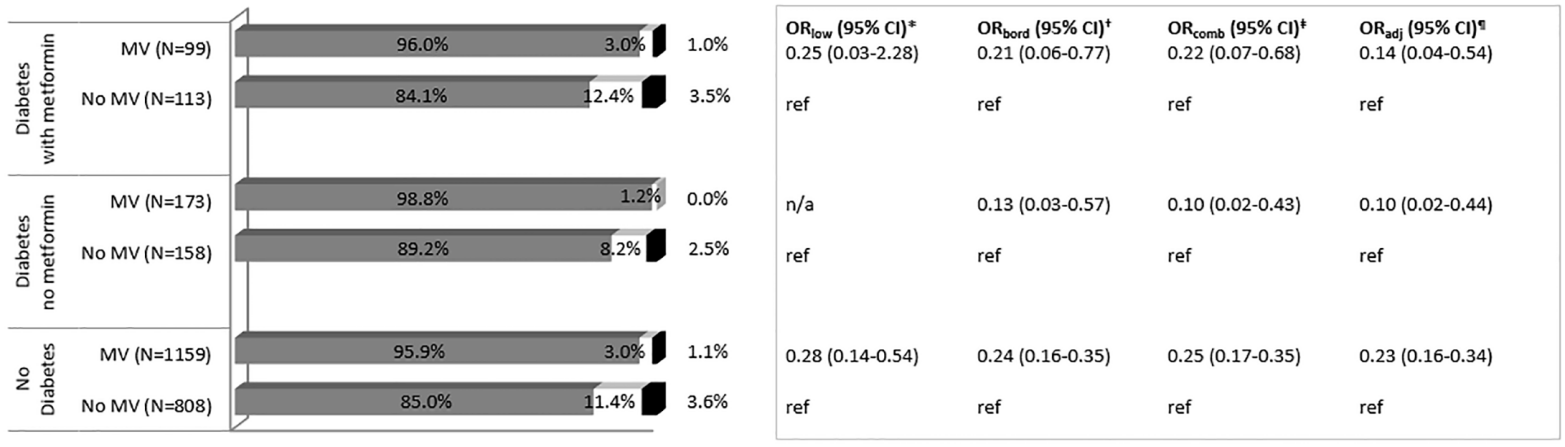
Prevalence of normal, borderline and low vitamin B12 concentrations, and its association with multivitamin use, stratified by diabetes/metformin status—REasons for Geographic And Racial Differences in Stroke, 2003–2007. Gray = % with normal serum B12 concentration; White = % with borderline serum B12 concentration; Black = % with low serum B12 concentration. *Unadjusted odds ratio (and 95% CI) for low B12 concentration and multivitamin use. †Unadjusted odds ratio (and 95% CI) for borderline B12 concentration (148–221 pmol/L vs. > 221 pmol/L) and multivitamin use. ‡Unadjusted odds ratio (and 95% CI) for combined low and borderline B12 concentration (≤221 pmol/L vs. > 221 pmol/L) and multivitamin use. ¶ Adjusted odds ratio (and 95% CI) for combined low and borderline B12 concentration (≤221 pmol/L vs. > 221 pmol/L) and multivitamin use, controlling for race, gender, geographic region, body mass index, alcohol use, smoking, insulin use, and age. OR = Odds Ratio; CI = Confidence Interval; MV = multivitamin; ref = Reference.

The multiple logistic regression analysis (Fig 4) showed that among participants with diabetes and taking metformin, multivitamin use was associated with a lower odds of low vitamin B12 concentration; however, this association was not statistically significant. The inverse association was, however, statistically significant among those with borderline B12 concentrations (OR_bord_ = 0.21; 95% CI = 0.06, 0.77), for combined low and borderline B12 concentrations (aOR_comb_ = 0.22; 95% CI = 0.07, 0.68), and for combined low and borderline concentrations in the adjusted model (aOR_adj_ = 0.14; 95% CI: 0.04–0.54). Additionally, a potentially protective association was also noted between multivitamin use and low vitamin B12 concentration (<148 pmol/L) among the other groups, i.e., those with diabetes but not taking metformin, and those without diabetes.

## Discussion

Our findings from a large sample of older adults in the United States suggest that multivitamin use can prevent low or borderline vitamin B12 concentrations among metformin users with diabetes. Participants with diabetes who used metformin had significantly lower serum B12 concentrations compared to participants with diabetes who did not use metformin. However, when we analyzed serum B12 concentrations categorically, it was less clear if multivitamin use was preventive of low B12 concentrations among metformin users. The association between multivitamin use and low vitamin B12 showed a strong inverse association, although not statistically significant, and is similar to the point estimate reported by Pflipsen et al. (2009) (OR = 0.31; 95% CI = 0.15, 0.63) [10]. Furthermore, our fully adjusted models found that multivitamins were strongly protective of B12 concentrations in both low and borderline groups among diabetic metformin users.

Our results showing diabetic metformin had significantly lower serum B12 concentrations compared to participants with diabetes who did not use metformin, confirms findings from previous studies that were mostly conducted in smaller samples [16]. Our results are also in agreement with previous studies where multivitamin use has been associated with improved serum B12 concentrations [23,26], and we have extended this finding to diabetic patients on metformin therapy.

Measurement of biochemical vitamin B12 deficiency is challenging. Consensus is lacking on the definition, and there is a high potential for misclassification. To have a comprehensive understanding of vitamin B12 deficiency, multiple biomarkers must be assessed. Recently NHANES proposed to include at least one biomarker (serum B12 or holotranscobalamin) and one functional biomarker (methylmalonic acid or total homocysteine) to have a better measure of vitamin B12 deficiency, and overcome limitations due to low sensitivity and specificity of individual biomarkers [21]. Population-based implications of such biomarker assessments have also been warranted.

It is currently unresolved whether metformin-induced vitamin B12 deficiency is best categorized as a cause of ‘clinical’, or alternatively, ‘subclinical (biochemical)’ B12 deficiency, and as such whether it should be treated. Carmel (2012) has stated that clinical B12 deficiency “arises from severe, long-term, and frequently irreversible malabsorption rooted in intrinsic-factor failure (best typified by pernicious anemia)” that guarantees progression towards clinical outcomes, such as anemia and neurologic symptoms if left untreated [29]. Given this definition, metformin-induced vitamin B12 deficiency shares several characteristics with clinical vitamin B12 deficiency, in that the vitamin B12 deficiency can become chronic depending on the dose and duration of exposure to metformin therapy, and that it can lead to anemia [8,30], and other neurologic symptoms [31,32].

Metformin use can lead to malabsorption of vitamin B12 as it is postulated that metformin interferes with calcium-dependent membrane action, affecting the uptake of the vitamin B12-intrinsic factor complex in the terminal ileum [33]. However, metformin-induced vitamin B12 deficiency also fits some characterizations of subclinical (or biochemical) B12 deficiency, in that the malabsorption is reversible upon discontinuance of metformin, that the malabsorption is less severe than that caused by pernicious anemia, and that the clinical symptoms are much less common. Regardless of its categorization, metformin-induced vitamin B12 deficiency is clearly different than “spontaneous” vitamin B12 deficiency, and should be considered as such in deciding how to treat it [12].

Screening and prevention techniques to treat and/or prevent metformin-induced B12 deficiency include annual vitamin B12 testing, monthly injections of vitamin B12 or large daily therapeutic doses (1000 mcg) of vitamin B12, prophylactically administered calcium carbonate (1.2 grams daily) [33], or, in severe cases, discontinuance of metformin therapy [12,24,31,34,35]. Multivitamin use is convenient, non-invasive, inexpensive, and generally effective in increasing serum B12 concentrations. Nevertheless, in our study, metformin users—the group at the highest risk for low serum B12 concentrations were actually less likely to have taken a multivitamin than those without diabetes.

A significant positive association between gastric acid inhibiting drugs (PPI and H2RA) and vitamin B12 deficiency has been reported recently [36]; however, we could not replicate this finding in our study sample (data not shown). Differences in subject characteristics and study methods may partly account for this discrepancy. Further analysis of this association is warranted.

Our findings are based on a cross-sectional analysis, using a biracial cohort originally recruited by REGARDS. While we controlled for several potential confounders, although residual confounding is still possible. For instance, multivitamin use may be a marker of healthy people [37,38]. However, there is also strong biological plausibility for a causal relationship between multivitamin supplementation and serum B12 concentrations [23–26,39], and it is difficult to imagine confounders that could explain the observed effect sizes. Lack of information on dose or duration of multivitamin use or of metformin use was a limitation. Multivitamins generally have about 6–25 μg of vitamin B12 per dose (25 μg per dose for those targeted to the elderly) and the median daily vitamin B12 dose that was reported in the Framingham Study cohort was 6 μg [39]. Although we did not have information on metformin dose. The usual dose of metformin given at the initiation of therapy is 500 mg/day, and rarely exceeds the maximum recommended dose of 2550 mg/day [40]. In terms of external validity, REGARDS study cohort may be healthier than the general population. The study had limited ability to consider other biomarkers (methylmalonic acid or homocysteine) to define B12 deficiency as these were not measured in the REGARDS; however, a consensus on the clinical definition of vitamin B12 deficiency is yet to be reached. Diabetes status was self-reported. However, previous studies showed that both validity and reliability of self-reported diabetes status among older adults are high [41–43]. We were unable to differentiate between type 1 and 2 diabetes; however, other studies using REGARDS cohort found that up to 95% of all diabetes cases are type 2 diabetes [44]. Our study focused on those aged 50 years or over, while the life expectancy is much shorter in type 1 diabetes patients [45].

There were several strengths to this study. The REGARDS study is a national cohort and has been extensively used in other epidemiological studies. Results from our study are exploratory and hypothesis generating. The REGARDS data allowed us to examine our study aims in an easy, feasible and cost-effective manner. Our study had a relatively large study sample compared to previous studies. Trained interviewers collected all data using a systematic protocol. Data on many demographic, health, and lifestyle factors were available to assess potential confounding. Measurement of multivitamin and metformin exposure was conducted by in-home pill bottle inspection and is reliable and valid.

Additional studies are needed to further examine the associations we reported, while including dose and duration of metformin exposure. Future research should also examine whether or not vitamin B12 therapy improves the health of persons taking metformin, especially as metformin use is common worldwide, and associated vitamin B12 deficiency. Vitamin B12 deficiency can be preventable, and treatable if identified early, in long-term metformin users. A longitudinal study should be done to assess how low and borderline B12 concentrations may be associated with clinical status and to assess the dose and duration of multivitamins necessary to modify the B12 concentrations among metformin users.
